# Stress Response of European Common Frog (*Rana temporaria*) Tadpoles to Bti Exposure in an Outdoor Pond Mesocosm

**DOI:** 10.1007/s00128-023-03708-6

**Published:** 2023-03-24

**Authors:** Verena Gerstle, Priyanka Solanki, Alessandro Manfrin, Sara Kolbenschlag, Carsten A. Brühl

**Affiliations:** iES Landau, Institute for Environmental Sciences, RPTU Kaiserslautern-Landau, Fortstraße 7, D-76829 Landau, Germany

**Keywords:** *Bacillus thuringiensis israelensis*, Mosquito control, Amphibians, GST, Protein carbonyl

## Abstract

The biocide *Bacillus thuringiensis* var. *israelensis* (Bti) is applied to wetlands to control nuisance by mosquitoes. Amphibians inhabiting these wetlands can be exposed to Bti multiple times, potentially inducing oxidative stress in developing tadpoles. For biochemical stress responses, ambient water temperature plays a key role. Therefore, we exposed tadpoles of the European common frog (*Rana temporaria*) three times to field-relevant doses of Bti in outdoor floodplain pond mesocosms (FPM) under natural environmental conditions. We sampled tadpoles after each Bti application over the course of a 51-day experiment (April to June 2021) and investigated the activity of the glutathione-S-transferase (GST) and protein carbonyl content as a measure for detoxification activity and oxidative damage. GST activity increased over the course of the experiment likely due to a general increase of water temperature. We did not observe an effect of Bti on either of the investigated biomarkers under natural ambient temperatures. However, Bti-induced effects may be concealed by the generally low water temperatures in our FPMs, particularly at the first application in April, when we expected the highest effect on the most sensitive early stage tadpoles. In light of the global climate change, temperature-related effects of pesticides and biocides on tadpoles should be carefully monitored - in particular since they are known as one of the factors driving the worldwide decline of amphibian populations.

**Supplementary Information:**

The online version contains supplementary material available at 10.1007/s00128-023-03708-6.

Insecticides are used in agricultural context for plant protection but also as biocides in vector control, for example against mosquitoes to combat malaria. Bio-insecticides which are assumed to have a lower environmental impact than synthetic insecticides applied in the past are increasingly used (Becker et al. 2018). This is particularly true in regions with mass emergence of floodwater mosquitoes, such as the Upper Rhine Valley in Southwest Germany. For over 40 years, products with the active ingredient *Bacillus thuringiensis* var. *israelensis* (Bti) are applied to these wetlands multiple times per year to control nuisance by mosquito bites (Becker 1997).

Bti is a bacterium which produces endotoxins during sporulation (Cry proteins; Margalith and Ben-Dov 2000). After ingestion, the Bti toxins bind to specific receptor sites in the gut of the organism which, under alkaline conditions, activates the toxin leading to a rapid death of the target organism (Bravo et al. 2007; Margalith and Ben-Dov 2000). Compared to synthetic insecticides, Bti toxins are considered to be environmentally friendly due to their low persistency (half-life 2–4 days or up to three weeks depending on the toxin and the environment; Tetreau et al. 2012) and specific mode of action towards target larvae of the dipteran suborder Nematocera. In Germany, viable Bti spores have to be sterilized using gamma-radiation prior to use in wetlands (Becker 2002), which prevents recycling of spores and proliferation in the field (Poulin et al. 2022). While effects on target organisms are well understood, recent studies shed light on the fact that Bti can affect non-target organisms in freshwater ecosystems, such as larvae of Chironomidae (e.g., Bordalo et al. 2020; Gerstle et al. 2023; Kästel et al. 2017), Coleoptera (Tudoran et al., 2021) and amphibian tadpoles (Allgeier et al. 2018; Gutierrez-Villagomez et al. 2021; Lajmanovich et al. 2015). For a detailed evaluation of the available environmental studies see also the review by Brühl et al. (2020).

Amphibians are rated the globally most threatened group of vertebrates (Munstermann et al. 2022; Stuart et al. 2004). The local decline of amphibian populations is a result of various impacts such as habitat loss due to climate change, habitat fragmentation and environmental contamination, diseases and invasive species as well as pesticides (Sparling et al. 2001; Stuart et al. 2004). In contrast to pesticides that end up in water bodies unintentionally, Bti is applied directly to the water surface (Becker 1997). Since many amphibian and mosquito species share breeding habitats in temporarily flooded wetlands, spawning of amphibians spatially and temporally coincides with Bti applications.

Due to the importance of amphibians for aquatic-terrestrial food webs and possible exposure to Bti, recent studies investigated direct effects of Bti exposure on amphibian tadpoles under laboratory conditions (as reviewed by Empey et al. 2021). A study from Argentina (Lajmanovich et al. 2015) reported reduced survival of Bti-exposed *Leptodactylus latrans* tadpoles, adverse effects on genotoxicity, erythrocyte nuclear abnormalities (ENAs) and an increase of stress biomarkers such as glutathione-S-transferase (GST) and catalase (CAT). The results of this study also raised concerns in Germany where Bti is applied on a large-scale in wetlands for decades and, so far, no evaluation of effects of the main formulations on native amphibians was performed. Hence, in a lab study in 2018, larvae of the European common frog (*Rana temporaria*) were exposed to Bti (Allgeier et al. 2018). *Rana temporaria* is the most common species in Europe belonging to the family Ranidae. In Germany, they breed in February and March in small stagnant (temporary) freshwater ponds and floodplains and their tadpoles develop for 8–10 weeks (Günther 1996), or faster depending on food resources and temperature (Günther 1996). Allgeier et al. (2018) recorded no effect on survival of *R. temporaria* tadpoles, but Bti resulted in increased activity levels of GST and glutathione-reductase (GR) indicating oxidative stress in tadpoles. In a follow-up study by Schweizer et al. (2019), evaluating histopathology and levels of B-esterases and Hsp70, no effects of Bti on stress responses of *R. temporaria* tadpoles were observed. These contrasting results may be attributed to the selection of different biomarkers and experimental conditions, such as the lower water temperature used in Schweizer et al. (2019) compared to Allgeier et al. (2018), i.e., 15°C vs. 18–24°C, respectively. Indeed, higher temperature induces thermal stress which can increase GST activity, while promoting oxidative damage to lipids, proteins, DNA and carbohydrates inside cells (Freitas et al. 2017). In both laboratory studies from Allgeier et al. (2018) and Schweizer et al. (2019), the limited capacity to implement environmental parameters and their range might have differently driven the results. In nature, realistic exposure scenarios do not only include diurnal temperature fluctuations and flooding events, but also limitations of food and space. In most laboratory studies, small groups of individuals are held in containers and supplied with highly nutritious fish food (Allgeier et al. 2018; Schweizer et al. 2019). These factors may influence body condition and, consequently, support energy-demanding processes like biochemical stress responses. Therefore, effects of Bti on *R. temporaria* tadpoles under realistic exposure scenarios in the field still remain unclear.

Because of the contrasting laboratory results, we assessed the direct effect of multiple Bti exposures on biochemical responses of *R. temporaria* tadpoles under natural climatic conditions in the field. We introduced early stage tadpoles (classified after Gosner 1960) to cages placed in twelve outdoor floodplain pond mesocosms (FPMs). Between April and June 2021, six FPMs were treated three times with the maximum recommended Bti field rate, the remaining six FPMs served as untreated controls. We sampled tadpoles 48 h after each of the three Bti applications, corresponding to early stage (GS 23), medium stage (GS 25) and late stage (GS 35–39) tadpoles, respectively. Because of the temperature increase over the course of the experiment from April to June, we expected a higher GST activity level in Bti-exposed tadpoles after the last application in summer, while minor effects would be observed at lower temperatures. On the other hand, as early stage tadpoles are hypothesized to be the most sensitive (Allgeier et al. 2018), we also expected a higher GST activity after the first application when tadpoles are in their early larval stage. As the increase in biomarker activity due to oxidative stress is likely induced by formation of reactive oxygen species (ROS), we analysed the protein carbonyl content as a measure for oxidative damage by ROS (Dalle-Donne et al. 2003). Assuming ROS formation after Bti exposure, we hypothesized that treated tadpoles show higher protein carbonylation compared to organisms from the control.

## Materials and Methods

The experiment took place in twelve constructed floodplain pond mesocosms (FPMs) at the Eußerthal Ecosystem Research Station (49° 15′ 14″ N, 7° 57′ 42″ E; EERES, University of Kaiserslautern-Landau) in the Palatinate Forest in Southwest Germany. The FPMs (23.5 × 7.5 m) are open to natural colonization from adjacent aquatic and terrestrial habitats since 2017 and thus considered to be established ecosystems in terms of flora and fauna (Stehle et al. 2022). They are 30 cm deep at the deepest point and gradually fade into a shallow water-land transition zone with a shore at one side. The FPMs can be flooded with stream water from the Sulzbach, an adjacent oligotrophic cold-water stream with minor anthropogenic influences in the upstream area (neither Bti or any mosquito control agent was applied in this region), until the terrestrial shore is completely under water (see Stehle et al. 2022 for further details).

In March 2021, six freshly laid egg clutches were collected from a pristine freshwater pond in close proximity to the FPMs (49° 15′ 17.1″ N, 7° 57′ 42.6″ E). Egg clutches were held separately in 10-L buckets filled with filtered (55 μm) stream water from the Sulzbach at outside temperatures. One week before test start, egg clutches were brought to the lab to hatch (T = 18 ± 2°C) to ensure we have enough tadpoles in GS 23 before start of the experiment. To avoid fungal growth, stream water in the buckets was renewed every day. Once hatched (two days before the first Bti application), the buckets containing the tadpoles were brought to the FPMs at outside temperatures to let them acclimatize for 24 h before transfer into the FPMs. To ensure high genetic variability among treatments, tadpoles hatched from six egg clutches were transferred into one tray.

One wooden cage (dimensions: 40 × 65 × 30 cm) with a 1-mm mesh was placed into the shallow water-land transition zone of each FPM with at least the bottom half of the cage permanently under water. Each cage was equipped with temperature-loggers (HOBO Pendant data logger, UA-002-64) fixed on the bottom of the cage, recording in 15-min intervals. To calculate the water temperature for each application (Figure S1), we used the mean temperature recorded by the loggers, starting from the day of application to sampling, covering the 48-h exposure period. Additionally, oxygen saturation, pH and water conductivity inside the cages were measured with a hand-held device (MultiLine Multi 3630, WTW Germany) once a week (Table S1). 24 h before the test start (i.e., first Bti application), 150 acclimatized tadpoles (GS 23) were randomly transferred into each cage. We introduced tadpoles instead of egg clutches to have equal numbers of individuals in the cage and to ensure that all introduced individuals were alive. Only freely swimming tadpoles with external gills were introduced (indicating Gosner stage 23). During the study period, we did not provide additional food for the tadpoles. However, due to flooding and water circulation through the mesh, detritus, algae and biofilm were sufficiently available.

**Fig. 1:**
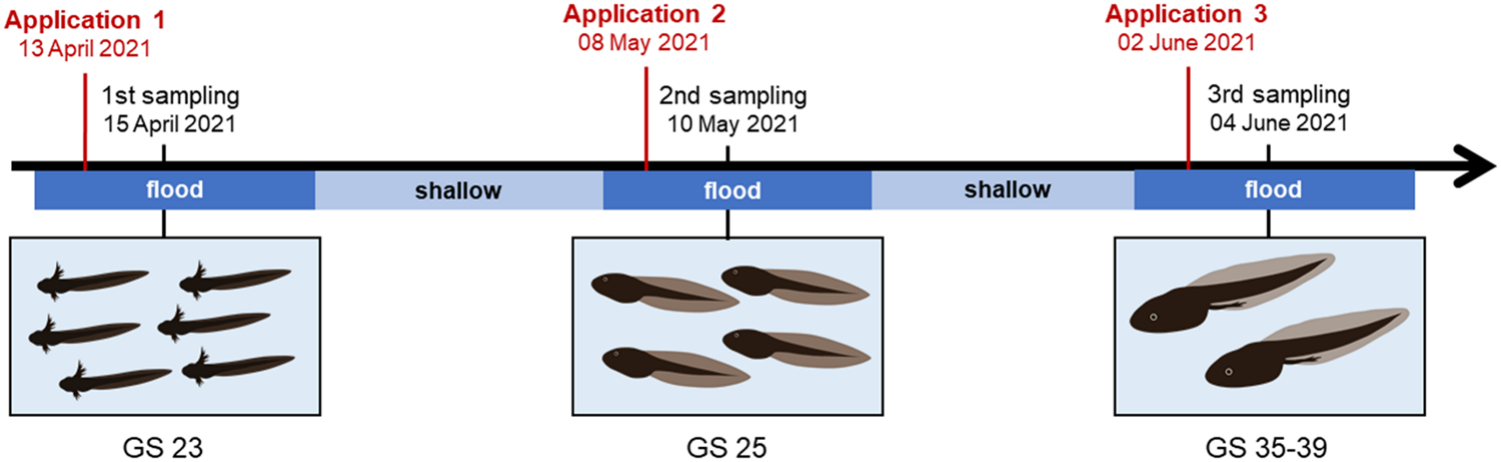
Schematic overview of the flooding, Bti application and samplings of *Rana temporaria* tadpoles from cages in the floodplain pond mesocosms, 48 h after the 1st, 2nd and 3rd Bti application at Gosner stages (GS) 23, 25 and 35–39

General procedure of flooding and Bti application are also described in detail in Gerstle et al. (2023) and Kolbenschlag et al. (2023). Regular Bti applications to the FPM system started in 2020, the year before this experiment was conducted. Since in Germany, only sterilized Bti spores are allowed (Becker 2002), we do not expect recycling of Bti spores as observed in the Camargue in France (Poulin et al. 2022). The maximum field rate (FR = 2.88 × 10^9^ international toxic units (ITU)/ha) of VectoBac WDG suspension (Valent BioSciences Corporation, Illinois, USA) was applied three times during the experiment (Fig. 1) to every second of the twelve FPMs using a knapsack sprayer (Prima 5, Gloria, Germany). In the Upper Rhine Valley, the maximum field rate of Bti is applied when late instar mosquito larvae are targeted or the water is deeper than 10 cm (Becker 1997). In this region, floodwater mosquitoes, whose larvae hatch when floodplains are inundated, are the main target organism. Therefore, the treatment of floodplains is strongly linked to rainfall- and snowmelt-induced flooding. Depending on the frequency of flooding events, Bti applications can be conducted in a weekly or bi-weekly interval, up to twelve times per year. Regular treatment of floodplains usually starts in March and last until late summer (Allgeier et al. 2018). Hence, also early stage tadpoles inhabiting these wetlands can be exposed multiple times during their development. To simulate a field-relevant application scenario, we linked Bti applications to controlled flooding periods of alternating 10-day flooding and 10-day shallow periods (see Fig. 1; exact dates varied according to weather conditions). 48 h after each Bti application, eight tadpoles of similar size were sampled from each cage by dip-netting, therefore a total of 288 tadpoles were used for biomarker assays. Individuals were euthanized in a buffered 0.1% MS-222 solution, shock frozen in liquid nitrogen and stored separately in 1.5-mL Eppendorf tubes at − 80°C until being used for biomarker analyses. In this study, we did not include body mass as an endpoint, since we focused on sampling tadpoles in the same Gosner stage (as done in Allgeier et al., 2018 and Schweizer et al., 2019), not individuals representing the average size in the cage. The study was terminated in mid-June (two weeks after the last sampling), before metamorphosis of the tadpoles, since in some FPMs the O_2_ saturation dropped under 20% and experimental conditions could not be maintained.

To verify the efficacy of Bti applications, we implemented a biotest using mosquito larvae as a reference organism, since currently there is no method available to quantify sterilized Bti-toxins in the environment. For details on the biotest see Gerstle et al. (2023) and Kolbenschlag et al. (2023).

We analysed GST, a phase II detoxifying enzyme involved in the antioxidant system (Steinberg 2012; Venturino and D’Angelo 2005). As a result of insufficient ROS defence by the antioxidant system inside the cells, proteins can be oxidized forming protein carbonyls (Dalle-Donne et al. 2003). Unlike ROS, protein carbonyls are stable making them a convenient biomarker for our study. Both biomarkers were expected to increase under stress (induced by Bti treatment) and were investigated spectrophotometrically using a multiplate reader (Synergy HT-I, BioTek, USA).

For the tissue homogenate, whole individuals were freeze-dried, weighed (Mettler Toledo, XA105 Dual Range, USA) and homogenized in RIPA lysis buffer (Thermo Scientific, USA) using a tissue lyzer (Retsch, MM 301, Germany) and metal beads. The tissue homogenate was used for both GST and protein carbonyl assays. To calculate the GST activity in nmol per mg protein per minute, total protein content was determined using a Micro BCA Protein Assay Kit (Thermo Scientific, USA) with bovine serum albumin (BSA) as standard. GST activity was measured following Habig et al. (1974) and Mingo et al. (2017), adapted for tissue homogenate. We used equine liver GST as an assay positive control. Protein carbonyl content was determined using a Protein Carbonyl Content Assay Kit (Sigma-Aldrich, USA). After derivatization of carbonyl groups with 2,4-dinitrophenyl-hydrazine (DNPH), protein contents in the derivatized samples were measured again using the same BCA Protein Content Assay Kit. Protein carbonyl was expressed in nmol carbonyl per mg protein.

Since negative GST activity levels are biologically impossible, negative values were considered zero (for GST, 9% of samples). Five values have been removed from the data set, due to methodological errors during the biomarker assays (see Table S2, S3). We performed linear mixed effect (LME) models to determine Bti effects on GST activity and protein carbonyl content using the *lme4* package (Bates et al. 2014; Pinheiro et al. 2017) for R (version 4.1.2; R Core Team, 2013). Using F-test-based backwards model selection (Zuur et al. 2009), we implemented treatment (control or Bti; n = 6) and application (1st, 2nd or 3rd application, also representing Gosner stages in course of the experiment) and their interaction as fixed effects in the final LME model. Since the water temperature did not vary significantly between the FPMs (see Figure S1, Table S1), we only used the three sequential applications to also describe temperature differences over the course of the experiment. We used FPM identity as random effect to account for multiple collection within each FPM (eight tadpoles for each FPM and application, resulting in a total of 288 tadpoles analysed). In case of a significant factor or factor interaction term, we used least-squares means (*lsmeans* package; Lenth, 2016) with FDR adjustment as a pairwise contrast post-hoc test to identify significant differences between groups. Response variables were log_10_-transformed to meet the model assumptions. Residual normality of the final model was checked graphically with quantile-quantile plots and heterogeneity with residuals versus fits plots (Zuur et al. 2009). The significance level for all analyses was set to *p* < 0.05. Plots were created using *ggplot2* (Wickham et al. 2016).

## Results and Discussion

**Fig. 2:**
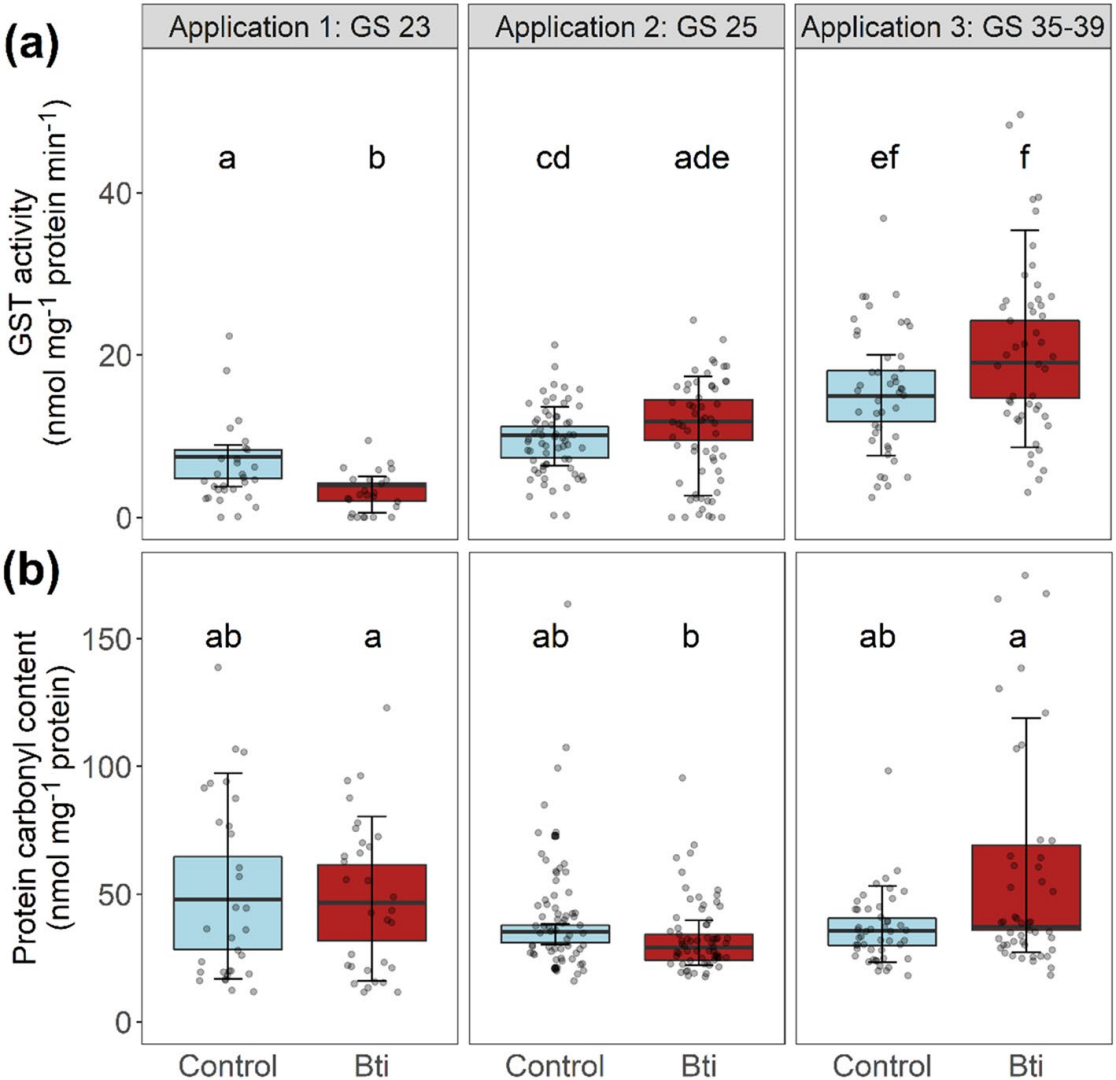
GST activity (**a**) and protein carbonyl content (**b**) in *R. temporaria* tadpoles from control and Bti-treated FPMs at Gosner stages (GS) 23, 25 and 35–39. Boxplots are based on mean values within the FPMs (n = 6), grey dots represent individual samples (N = 288 for each biomarker). Lower and upper box boundaries show 25th and 75th percentiles, respectively, line inside the box show the median. Whiskers and black dots show the variability outside the lower and upper quartiles. Groups that do not share a common letter differ statistically significant (based on least-squares means)

**Fig. 3:**
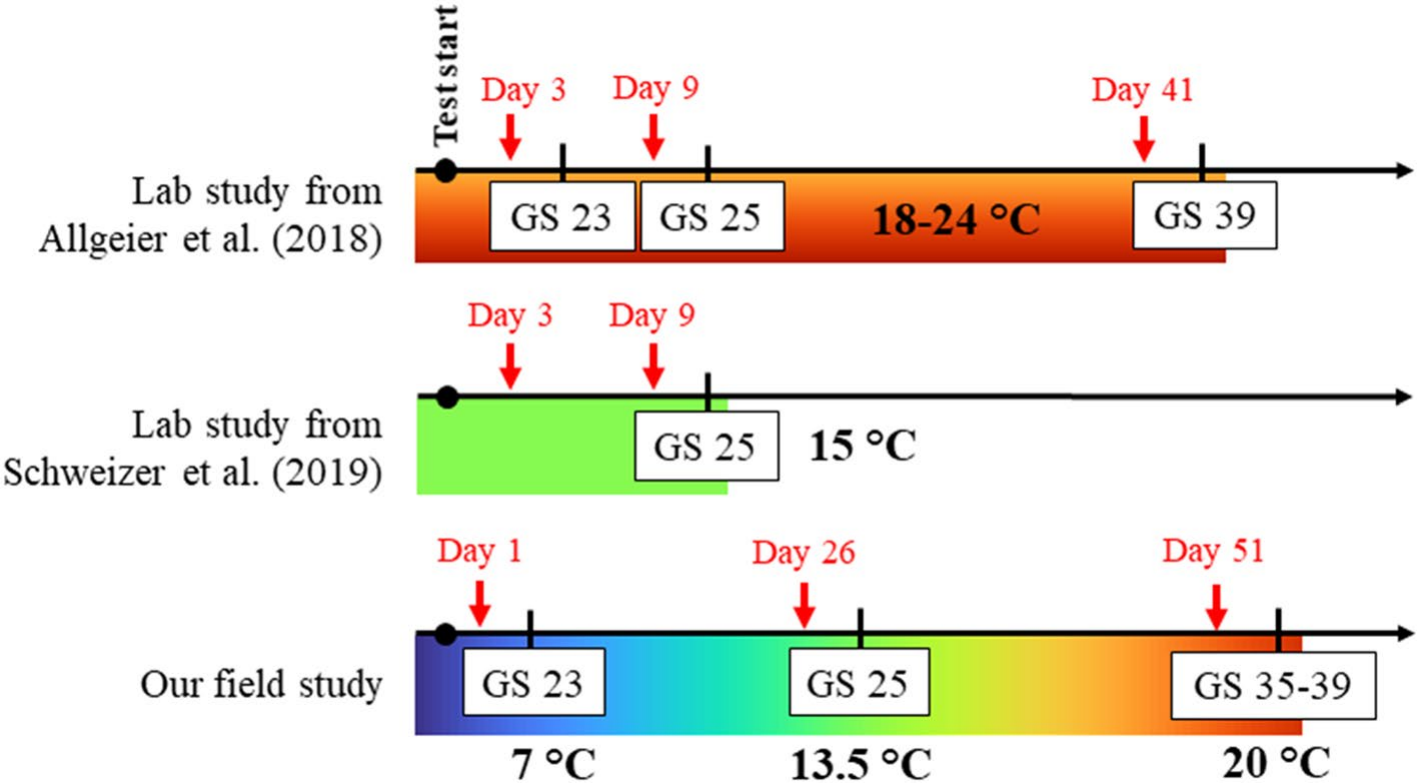
Comparison of water temperatures over the course of the three discussed studies. Red arrows represent Bti applications, black lines indicate tadpole samplings (48 h after Bti application) with information on Gosner stages (GS) and mean temperature (printed in bold)

We measured lower (52%) mean GST activity levels in Bti-treated tadpoles after the first application compared to the control, while results (although not significant) suggest a pattern of higher levels in Bti-treated tadpoles after applications 2 and 3, 17% and 38%, respectively (Figure 2 a, Table 1, Table S2, S4). Also, mean GST activity levels in tadpoles increased over the course of the experiment after each application (here representative for Gosner stage and water temperature), with increasing water temperatures, which were on average 7, 13.5 and 20°C for application 1, 2 and 3, respectively (Figure 3, Figure S1). In aquatic organisms, GST activity levels have been reported to react sensitively to thermal stress as shown in saltwater fish (Madeira et al., 2013) and neotropical tadpoles (Freitas et al., 2017; Freitas and Almeida, 2016). Since abiotic environmental variables like high water temperature can amplify toxic effects of Bti in chironomid larvae (Charbonneau et al., 1994), we also expected an increase in GST activity in Bti-exposed tadpoles, especially with increasing temperature, which was not recorded in our experiment.

**Table 1:** Effect of Bti treatment and number of applications (as proxy for the temporal aspect, i.e., increase in water temperature and deveolmental stage of tadpoles) on GST activity and protein carbonyl content in R.* temporaria* tadpoles

	numDF	denDF	*F* value	*p* value
*GST activity*				
Treatment	1	10	0.483	0.503
Application	2	270	69.218	**< 0.001**
Treatment × Application	2	270	10.100	**< 0.001**
*Protein carbonyl content*				
Treatment	1	10	0.006	0.938
Application	2	269	6.046	**< 0.003**
Treatment × Application	2	269	9.007	**< 0.002**

Numerator degrees of freedom (numDF), denominator degrees of freedom (denDF), F values and p values are shown, statistically significant p values are printed in bold

Our results from the field are comparable to the findings of Schweizer et al. (2019), who did not record any Bti-induced difference in stress-related biomarker levels in *R. temporaria* tadpoles at 15°C. We observed a similar temperature of 13.5°C after the second Bti application in May (Figure S1, Table S1). However, we expected the highest effect on sensitive early stage tadpoles, which we sampled in mid-April at a significantly lower temperature (7°C), compared to Allgeier et al. (2018) and Schweizer et al. (2018) (for comparison see Fig. 3). The cold-water scenario at application 1 may be due to the fact that we experienced an exceptionally cold April in 2021 compared to the previous years (Deutscher Wetterdienst, 2023), potentially buffering the effects on early stage tadpoles. Additionally, our test facility is located in a temperate forest with generally lower temperatures compared to water bodies in the Upper Rhine Valley, where Bti is applied. Although Schweizer et al. (2019) claimed that the effects observed by Allgeier et al. (2018) were possibly due to the high water temperatures (18–24°C), we measured peak temperatures of up to 20°C at the end of April (Table S1). In spring, water temperatures of up to 25°C in ponds in our regions are common, especially in ponds located in lowlands of the Upper Rhine Valley (Adams et al. 2021), which can be considerably warmer than our FPMs. Therefore, it is possible that early and medium stage tadpoles are temporarily exposed to such temperature conditions used by Allgeier et al. (2018). Despite the lack of the effect we expected in early stage tadpoles, we also assumed a significantly higher Bti-induced effect at warmer temperatures, which was not recorded after the third application at 20°C. We only observed a slightly increased GST activity in late stage tadpoles (Fig. 2a). However, a possible effect of warmer exposure conditions could be reduced due to higher developmental stages of tadpoles which are expected to be less sensitive. In other words, if applications of Bti in early spring coincide with peak temperatures of 20°C, oxidative stress in early stage *R. temporaria* tadpoles is more likely to happen as reported in Allgeier et al. (2018). An increase of cellular responses to xenobiotics is linked to a higher cost of energy (Steinberg 2012). In turn, this may impair tadpoles’ behavior and development, possibly affecting amphibian populations at a later stage (Monaghan et al. 2009). However, this was not observed under the comparably cold temperatures experienced during our experiment.

Similar to GST activity levels, there was no significant Bti-induced effect on protein carbonyl contents due to oxidative stress (Fig. 2b; Table 1, Table S3, S5). In contrast, Gutierrez-Villagomez et al. (2021) exposed tadpoles of two North American frog species (*Lithobates sylvaticus* and *Anaxyrus americanus*) to two different Bti formulations in a chronic exposure laboratory experiment. Authors did not record a significant change in stress response in a dose-dependent pattern, but they observed modifications in the intestine microbiota as well as an overexpression of the genes *cyp1a* and *sod* in tails of *L. sylvaticus*. Overexpression of *cyp1a* and *sod* may indicate detoxification processes and oxidative stress, but this pattern was not observed in *A. americanus* tadpoles. The *sod* gene expresses an enzyme which is responsible for destroying radicals, such as ROS. In our experiment, we assumed ROS formation in Bti-exposed tadpoles resulting in protein damage, i.e., increased protein carbonylation. However, in our scenario, Bti did not have an effect on protein carbonyl content. Our findings suggest insignificant levels of ROS formation; thus, we assume that Bti does not affect proteins in *R. temporaria* tadpoles under the tested conditions.

This is the first study investigating oxidative stress response in tadpoles exposed to Bti under natural environmental conditions. Our results show no significant increase of biochemical biomarkers as response to stress from Bti treatment. However, we are cautious to not generalise our findings to different environmental conditions, species or different developmental stages of such a worldwide threatened vertebrate group like amphibians. During our experiment, exceptional cold temperatures in early spring prevented our pond mesocosm from being an accurate representation of wetlands in the Upper Rhine Valley, where Bti is applied and water temperatures can be considerably warmer. Additionally, with global climate change suggesting increased water temperatures in the future, it is likely that amphibians in temperate regions will face higher water temperatures during their whole aquatic development (Noyes et al. 2009). Although our results do not suggest an effect of temperature on the toxicity of Bti, natural temperature fluctuations should be considered in biocide and pesticide toxicity tests (Baier et al. 2016; Leeb et al. 2022), especially of substances intentionally applied to amphibian-rich wetlands. Indirect effects of Bti on food webs were not addressed in this study. However, recent investigations (e.g. Gutierrez-Villagomez et al., 2021; McKie et al., 2023) revealed that Bti can have implications on the trophic structure in aquatic ecosystems, potentially affecting amphibians. This highlights the importance of further research on indirect effects of Bti on aquatic food webs.

## Supplementary Information

Below is the link to the electronic supplementary material.

Supplementary material 1 (DOCX 399.8 kb)

## Abbreviations

Bti: *Bacillus thuringiensis* var. *israelensis*
CAT: Catalase
ENAs: Erythrocyte nuclear abnormalities
EERES: Eußerthal Ecosystem Research Station
FPM: Floodplain pond mesocosm
FR: Field rate
GST: Glutathione-S-transferase
ITU: International toxic unit
LME: Linear mixed effect model
ROS: Reactive oxygen species
