# Supplementary material for: Stress Response of European Common Frog (Rana temporaria) Tadpoles to Bti Exposure in an Outdoor Pond Mesocosm
Source: Bull Environ Contam Toxicol. 2023 Mar 24;110(4):70. doi: 10.1007/s00128-023-03708-6 (PMC10036417; doi:10.1007/s00128-023-03708-6)
Supplement: Supplementary file 1 — Supplementary material 1 (DOCX 399.8 kb) [file 128_2023_3708_MOESM1_ESM.docx]

# Supplementary Information


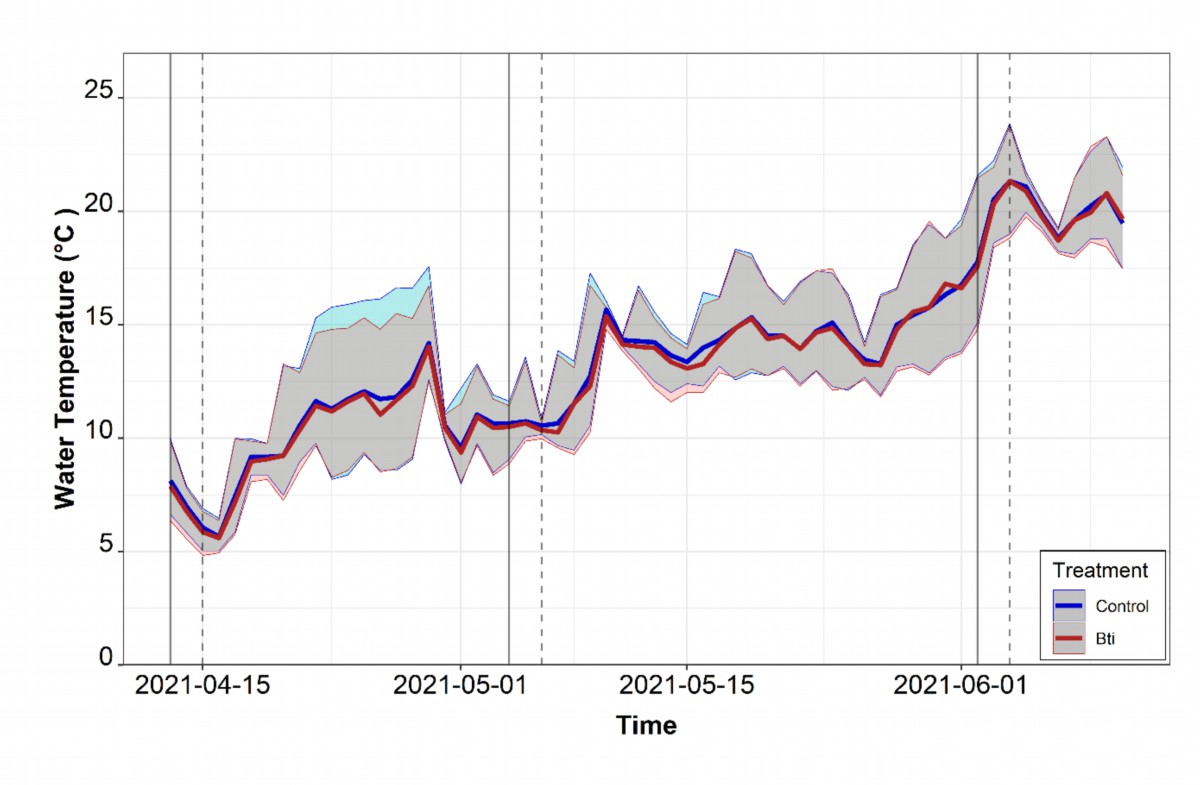


Figure S 1: Water temperature in control (blue; n=6) and Bti-treated FPMs (red; n=6) over the course of the experiment. Shown are the median temperatures with lower and upper quartiles and the date of the Bti application (solid line) and tadpole samplings (dashed line).

Table S 1: Environmental parameters measured inside the cages in each FPM. Sampling dates are printed in bold.

| Date | FPM | Time | Water level (cm) | pH | Conductivity (µS/cm) | O_2_  (mg/L) | O_2_  (%) | Temperature  (°C) |
| --- | --- | --- | --- | --- | --- | --- | --- | --- |
| 2021 Apr 13 | 1 | 12:33 | 53 | 7.62 | 99.9 | 10.48 | 86.8 | 6.8 |
|  | 2 | 12:31 | 52 | 7.74 | 124.4 | 11.19 | 92.9 | 6.9 |
|  | 3 | 12:29 | 51 | 7.75 | 125.5 | 11.29 | 95.0 | 7.5 |
|  | 4 | 12:27 | 52 | 7.58 | 126.2 | 11.16 | 94.0 | 7.6 |
|  | 5 | 12:24 | 51 | 7.72 | 98.3 | 11.34 | 95.9 | 7.8 |
|  | 6 | 12:22 | 52 | 7.68 | 124.2 | 11.22 | 94.8 | 7.7 |
|  | 7 | 12:20 | 52 | 7.55 | 115.5 | 10.90 | 90.6 | 7.1 |
|  | 8 | 12:44 | 52 | 7.48 | 104.6 | 11.41 | 95.5 | 7.3 |
|  | 9 | 12:43 | 51 | 7.74 | 97.8 | 11.11 | 93.5 | 7.5 |
|  | 10 | 12:42 | 52 | 7.72 | 139.2 | 11.10 | 91.1 | 6.6 |
|  | 11 | 12:41 | 52 | 7.72 | 122.5 | 10.32 | 84.9 | 6.7 |
|  | 12 | 12:39 | 48 | 7.68 | 152.1 | 10.78 | 87.9 | 6.5 |
| **2021 Apr 15** | 1 | 10:27 | 53 | 7.68 | 106.2 | 11.45 | 87.9 | 3.9 |
|  | 2 | 10:25 | 52 | 7.78 | 136.6 | 11.91 | 91.8 | 4.0 |
|  | 3 | 10:23 | 51 | 7.78 | 136.3 | 12.05 | 92.2 | 3.7 |
|  | 4 | 10:21 | 52 | 7.68 | 130.0 | 11.70 | 89.6 | 3.6 |
|  | 5 | 10:19 | 50 | 7.86 | 105.7 | 11.99 | 92.0 | 4.0 |
|  | 6 | 10:17 | 50 | 7.76 | 132.6 | 11.62 | 87.6 | 3.2 |
|  | 7 | 10:15 | 52 | 7.71 | 123.2 | 12.02 | 92.1 | 3.9 |
|  | 8 | 10:13 | 52 | 7.82 | 109.8 | 11.61 | 88.4 | 3.8 |
|  | 9 | 10:11 | 50 | 7.82 | 105.6 | 11.72 | 88.7 | 3.1 |
|  | 10 | 10:09 | 52 | 7.95 | 145.7 | 12.21 | 93.7 | 3.9 |
|  | 11 | 10:07 | 52 | 7.89 | 138.3 | 11.38 | 87.8 | 4.1 |
|  | 12 | 10:04 | 48 | 8.08 | 157.2 | 11.86 | 90.5 | 3.7 |
| 2021 Apr 22 | 1 | 15:32 | 30 | 7.66 | 132.5 | 10.99 | 114.0 | 16.2 |
|  | 2 | 15:29 | 30 | 8.65 | 150.6 | 15.13 | 156.7 | 16.1 |
|  | 3 | 15:27 | 30 | 8.14 | 156.1 | 13.32 | 140.6 | 17.6 |
|  | 4 | 15:25 | 30 | 8.13 | 140.4 | 12.36 | 131.0 | 17.3 |
|  | 5 | 15:22 | 31 | 8.46 | 115.9 | 13.37 | 142.5 | 17.5 |
|  | 6 | 15:20 | 30 | 8.12 | 152.5 | 13.23 | 139.6 | 17.1 |
|  | 7 | 15:18 | 31 | 8.1 | 154.2 | 13.48 | 143.5 | 17.5 |
|  | 8 | 15:16 | 30 | 7.91 | 122.3 | 13.03 | 133.0 | 15.4 |
|  | 9 | 15:13 | 31 | 8.45 | 112.1 | 14.14 | 148.5 | 16.8 |
|  | 10 | 15:11 | 30 | 7.86 | 184.5 | 13.01 | 132.4 | 15.3 |
|  | 11 | 15:09 | 31 | 7.93 | 179.7 | 12.07 | 128.0 | 17.2 |
|  | 12 | 15:06 | 31 | 7.31 | 173.2 | 11.50 | 115.6 | 15.1 |
| 2021 Apr 29 | 1 | 16:10 | 29 | 8.23 | 124.8 | 11.35 | 126.6 | 18.7 |
|  | 2 | 16:05 | 29 | 9.61 | 151.0 | 16.79 | 184.2 | 18.8 |
|  | 3 | 16:03 | 29 | 8.76 | 168.0 | 13.79 | 152.7 | 18.9 |
|  | 4 | 16:01 | 29 | 8.87 | 153.6 | 13.04 | 148.2 | 19.8 |
|  | 5 | 15:59 | 30 | 9.12 | 112.3 | 13.70 | 154.7 | 19.4 |
|  | 6 | 15:56 | 28 | 8.81 | 164.1 | 11.94 | 135.0 | 19.4 |
|  | 7 | 15:54 | 30 | 8.88 | 140.4 | 13.27 | 148.6 | 18.8 |
|  | 8 | 15:51 | 29 | 9.08 | 122.4 | 13.38 | 147.0 | 18.4 |
|  | 9 | 15:49 | 30 | 9.61 | 136.3 | 16.49 | 183.6 | 19.0 |
|  | 10 | 15:46 | 30 | 8.97 | 156.9 | 11.64 | 126.0 | 17.7 |
|  | 11 | 15:44 | 30 | 7.8 | 197.7 | 10.91 | 120.8 | 18.8 |
|  | 12 | 15:41 | 30 | 7.31 | 185.4 | 10.04 | 108.8 | 17.6 |
| **2021 May 10** | 1 | 11:07 | 52 | 7.58 | 97.3 | 9.72 | 87.0 | 9.5 |
|  | 2 | 11:05 | 50 | 7.94 | 129.9 | 10.73 | 93.8 | 8.9 |
|  | 3 | 11:03 | 52 | 7.83 | 131.2 | 10.73 | 95.1 | 9.1 |
|  | 4 | 10:59 | 51 | 7.7 | 118.6 | 10.76 | 95.6 | 9.2 |
|  | 5 | 10:57 | 51 | 7.64 | 102.3 | 10.32 | 92.5 | 9.5 |
|  | 6 | 10:54 | 49 | 7.62 | 122.9 | 10.65 | 94.2 | 9.0 |
|  | 7 | 10:52 | 52 | 7.56 | 122.6 | 9.97 | 87.7 | 8.8 |
|  | 8 | 10:49 | 51 | 7.66 | 107.7 | 10.59 | 92.6 | 8.5 |
|  | 9 | 10:47 | 50 | 7.79 | 107.3 | 10.68 | 92.2 | 8.0 |
|  | 10 | 10:45 | 52 | 7.78 | 133.8 | 10.34 | 88.3 | 7.7 |
|  | 11 | 10:43 | 53 | 7.74 | 136.1 | 9.55 | 81.7 | 7.8 |
|  | 12 | 10:41 | 52 | 7.72 | 141.7 | 9.69 | 83.0 | 7.8 |
| 2021 May 17 | 1 | 16:32 | 30 | 7.37 | 123.6 | 9.98 | 107.2 | 17.3 |
|  | 2 | 16:30 | 30 | 7.22 | 126.3 | 14.08 | 151.0 | 17.0 |
|  | 3 | 16:27 | 30 | 7.78 | 150.1 | 11.99 | 129.7 | 17.5 |
|  | 4 | 16:25 | 31 | 7.85 | 120.1 | 12.52 | 136.6 | 17.9 |
|  | 5 | 16:23 | 30 | 7.85 | 98.2 | 13.97 | 151.0 | 17.5 |
|  | 6 | 16:21 | 31 | 7.50 | 127.3 | 12.12 | 130.7 | 17.3 |
|  | 7 | 16:19 | 29 | 7.40 | 143.7 | 11.57 | 124.9 | 17.4 |
|  | 8 | 16:16 | 31 | 7.40 | 109.7 | 10.64 | 115.2 | 17.0 |
|  | 9 | 16:14 | 29 | 7.37 | 132.7 | 12.44 | 132.6 | 17.1 |
|  | 10 | 16:11 | 30 | 7.31 | 211.0 | 7.83 | 83.2 | 16.6 |
|  | 11 | 16:08 | 32 | 7.43 | 230.0 | 10.08 | 108.2 | 17.1 |
|  | 12 | 16:06 | 30 | 7.41 | 187.1 | 10.30 | 109.2 | 16.5 |
| 2021 May 25 | 1 | 13:25 | 33 | 7.54 | 93.7 | 10.60 | 103.3 | 12.0 |
|  | 2 | 13:23 | 32 | 7.58 | 151.7 | 9.05 | 85.7 | 12.1 |
|  | 3 | 13:21 | 32 | 7.97 | 151.4 | 10.71 | 101.9 | 12.0 |
|  | 4 | 13:19 | 31 | 8.49 | 105.3 | 11.91 | 113.9 | 12.4 |
|  | 5 | 13:17 | 32 | 8.44 | 86.7 | 12.92 | 123.4 | 12.3 |
|  | 6 | 13:15 | 32 | 7.75 | 132.7 | 10.76 | 102.5 | 12.3 |
|  | 7 | 13:12 | 32 | 7.6 | 116.1 | 11.68 | 111.5 | 12.3 |
|  | 8 | 13:10 | 32 | 7.75 | 90.7 | 10.45 | 99.4 | 12.2 |
|  | 9 | 13:08 | 32 | 8.21 | 127.4 | 12.03 | 114.0 | 11.9 |
|  | 10 | 13:06 | 34 | 7.65 | 148.1 | 10.29 | 96.7 | 11.9 |
|  | 11 | 13:04 | 35 | 7.50 | 189.8 | 8.95 | 84.7 | 12.0 |
|  | 12 | 13:02 | 33 | 6.93 | 170.2 | 9.98 | 93.3 | 12.2 |
| **2021 June 04** | 1 | 10:59 | 51 | 7.28 | 102.9 | 5.91 | 66.7 | 20.4 |
|  | 2 | 10:57 | 50 | 7.48 | 147.9 | 6.83 | 76.2 | 19.8 |
|  | 3 | 10:55 | 52 | 7.44 | 142.6 | 7.33 | 81.8 | 19.7 |
|  | 4 | 10:53 | 51 | 7.37 | 122.3 | 6.78 | 75.4 | 20.2 |
|  | 5 | 10:51 | 51 | 7.55 | 101.0 | 8.44 | 94.3 | 20.0 |
|  | 6 | 10:49 | 50 | 7.49 | 115.6 | 9.64 | 106.2 | 19.3 |
|  | 7 | 10:47 | 51 | 7.56 | 124.9 | 8.77 | 96.0 | 19.0 |
|  | 8 | 10:45 | 51 | 7.52 | 103.3 | 9.42 | 103.4 | 19.1 |
|  | 9 | 10:43 | 50 | 7.42 | 107.2 | 8.16 | 89.1 | 18.8 |
|  | 10 | 10:41 | 52 | 7.62 | 135.7 | 8.41 | 91.1 | 18.4 |
|  | 11 | 10:39 | 53 | 7.64 | 134.8 | 8.27 | 89.7 | 18.4 |
|  | 12 | 10:37 | 52 | 7.49 | 137.9 | 7.02 | 76.3 | 18.6 |
|  |  |  |  |  |  |  |  |  |

Table S 2: GST activity (mean ± SD; nmol/mg protein min) in *Rana temporaria* tadpoles sampled 48h after each Bti application.

| **FPM** | **Treatment** | **Application 1** | | | | | **Application 2** | | | | **Application 3** | | | |  |
| --- | --- | --- | --- | --- | --- | --- | --- | --- | --- | --- | --- | --- | --- | --- | --- |
|  |  | **GST activity** | | | | **n** | **GST activity** | | | **n** | **GST activity** | | | **n** |  |
| 1 | Control | 4.03 | ± | 1.89 | 8 | | 6.33 | ± | 3.40 | 8 | 16.06 | ± | 9.05 | 8 | |
| 2 | Bti | 3.97 | ± | 2.16 | 7 | | 9.50 | ± | 8.76 | 8 | 23.13 | ± | 4.77 | 8 | |
| 3 | Control | 7.94 | ± | 3.63 | 7 | | 6.64 | ± | 4.78 | 8 | 11.11 | ± | 8.20 | 8 | |
| 4 | Bti | 0.57 | ± | 0.93 | 8 | | 2.67 | ± | 2.65 | 8 | 14.53 | ± | 9.35 | 8 | |
| 5 | Control | 7.00 | ± | 6.35 | 8 | | 11.20 | ± | 3.47 | 8 | 18.71 | ± | 3.36 | 8 | |
| 6 | Bti | 3.87 | ± | 3.27 | 8 | | 14.04 | ± | 1.96 | 8 | 24.54 | ± | 6.91 | 8 | |
| 7 | Control | 3.76 | ± | 6.13 | 8 | | 13.55 | ± | 4.66 | 8 | 13.82 | ± | 8.11 | 8 | |
| 8 | Bti | 1.41 | ± | 1.75 | 8 | | 17.31 | ± | 2.89 | 8 | 35.36 | ± | 10.34 | 8 | |
| 9 | Control | 8.38 | ± | 2.80 | 8 | | 11.05 | ± | 3.72 | 8 | 20.02 | ± | 9.45 | 8 | |
| 10 | Bti | 4.28 | ± | 5.56 | 8 | | 9.52 | ± | 3.67 | 8 | 14.97 | ± | 5.71 | 8 | |
| 11 | Control | 8.93 | ± | 4.25 | 8 | | 9.23 | ± | 3.08 | 8 | 7.61 | ± | 5.22 | 8 | |
| 12 | Bti | 4.99 | ± | 4.99 | 8 | | 14.64 | ± | 3.72 | 8 | 8.60 | ± | 4.95 | 8 | |

| **FPM** | **Treatment** | **Application 1** | | | | **Application 2** | | | | **Application 3** | | | |
| --- | --- | --- | --- | --- | --- | --- | --- | --- | --- | --- | --- | --- | --- |
|  |  | **Protein carbonyl** | | | **n** | **Protein carbonyl** | | | **n** | **Protein carbonyl** | | | **n** |
| 1 | Control | 24.13 | ± | 6.63 | 7 | 36.50 | ± | 7.90 | 8 | 53.16 | ± | 19.69 | 8 |
| 2 | Bti | 28.77 | ± | 11.21 | 8 | 22.43 | ± | 2.61 | 8 | 35.95 | ± | 10.61 | 8 |
| 3 | Control | 16.71 | ± | 3.11 | 8 | 20.93 | ± | 3.07 | 8 | 28.17 | ± | 5.30 | 8 |
| 4 | Bti | 16.02 | ± | 4.81 | 7 | 22.27 | ± | 3.71 | 8 | 79.44 | ± | 51.22 | 7 |
| 5 | Control | 97.29 | ± | 20.05 | 8 | 72.85 | ± | 38.02 | 8 | 34.62 | ± | 4.90 | 8 |
| 6 | Bti | 80.33 | ± | 21.45 | 8 | 39.70 | ± | 6.97 | 8 | 35.79 | ± | 12.61 | 8 |
| 7 | Control | 55.44 | ± | 20.54 | 8 | 33.89 | ± | 6.36 | 8 | 23.45 | ± | 4.61 | 8 |
| 8 | Bti | 64.61 | ± | 17.19 | 8 | 29.56 | ± | 3.87 | 8 | 27.34 | ± | 10.60 | 8 |
| 9 | Control | 67.71 | ± | 27.18 | 8 | 30.20 | ± | 6.85 | 8 | 36.93 | ± | 8.58 | 8 |
| 10 | Bti | 52.25 | ± | 21.42 | 8 | 35.90 | ± | 16.19 | 8 | 118.91 | ± | 38.65 | 8 |
| 11 | Control | 40.46 | ± | 12.23 | 8 | 38.18 | ± | 13.04 | 8 | 41.66 | ± | 11.22 | 8 |
| 12 | Bti | 40.93 | ± | 14.35 | 8 | 28.89 | ± | 4.41 | 8 | 38.63 | ± | 7.63 | 8 |

Table S 3: Protein carbonyls (mean ± SD; nmol/mg protein) in *Rana temporaria* tadpoles sampled 48h after each Bti application.

Table S 4: Comparison of groups using least squares-means on log-transformed GST activity levels with standard errors (SE), degrees of freedom (df) and T ratio. Significant differences are printed in bold.

| Comparison | Estimate | SE | df | T ratio | P value |
| --- | --- | --- | --- | --- | --- |
| Bti 1 - Control 1 | -0.347 | 0.112 | 10 | -3.093 | **0.019** |
| Bti 1 - Bti 2 | -0.540 | 0.076 | 270 | -7.064 | **<0.001** |
| Bti 1 - Control 2 | -0.617 | 0.103 | 10 | -5.994 | **<0.001** |
| Bti 1 - Bti 3 | -0.897 | 0.077 | 270 | -11.584 | **<0.001** |
| Bti 1 - Control 3 | -0.738 | 0.105 | 10 | -7.014 | **<0.001** |
| Control 1 - Bti 2 | -0.193 | 0.103 | 10 | -1.875 | 0.104 |
| Control 1 - Control 2 | -0.270 | 0.076 | 270 | -3.524 | **<0.001** |
| Control 1 - Bti 3 | -0.549 | 0.105 | 10 | -5.224 | **<0.001** |
| Control 1 - Control 3 | -0.390 | 0.077 | 270 | -5.043 | **<0.001** |
| Bti 2 - Control 2 | -0.077 | 0.093 | 10 | -0.827 | 0.427 |
| Bti 2 - Bti 3 | -0.357 | 0.063 | 270 | -5.659 | **<0.001** |
| Bti 2 - Control 3 | -0.197 | 0.095 | 10 | -2.077 | 0.081 |
| Control 2 - Bti 3 | -0.280 | 0.095 | 10 | -2.945 | **0.022** |
| Control 2 - Control 3 | -0.121 | 0.063 | 270 | -1.919 | 0.076 |
| Bti 3 - Control 3 | 0.159 | 0.098 | 10 | 1.631 | 0.144 |

Table S 5: Comparison of groups using least squares-means on log-transformed GST activity levels with standard errors (SE), degrees of freedom (df) and T ratio. Significant differences are printed in bold.

| Comparison | Estimate | SE | df | T ratio | P value |
| --- | --- | --- | --- | --- | --- |
| Bti 1 - Control 1 | 0.043 | 0.086 | 10 | 0.502 | 0.672 |
| Bti 1 - Bti 2 | 0.163 | 0.044 | 269 | 3.738 | **0.002** |
| Bti 1 - Control 2 | 0.068 | 0.083 | 10 | 0.827 | 0.642 |
| Bti 1 - Bti 3 | -0.012 | 0.044 | 269 | -0.281 | 0.779 |
| Bti 1 - Control 3 | 0.104 | 0.083 | 10 | 1.250 | 0.550 |
| Control 1 - Bti 2 | 0.119 | 0.083 | 10 | 1.445 | 0.537 |
| Control 1 - Control 2 | 0.025 | 0.043 | 269 | 0.572 | 0.655 |
| Control 1 - Bti 3 | -0.056 | 0.083 | 10 | -0.667 | 0.650 |
| Control 1 - Control 3 | 0.061 | 0.044 | 269 | 1.394 | 0.537 |
| Bti 2 - Control 2 | -0.094 | 0.078 | 10 | -1.203 | 0.550 |
| Bti 2 - Bti 3 | -0.175 | 0.036 | 269 | -4.913 | **<0.001** |
| Bti 2 - Control 3 | -0.058 | 0.079 | 10 | -0.735 | 0.650 |
| Control 2 - Bti 3 | -0.081 | 0.079 | 10 | -1.014 | 0.558 |
| Control 2 - Control 3 | 0.036 | 0.035 | 269 | 1.017 | 0.558 |
| Bti 3 - Control 3 | 0.117 | 0.080 | 10 | 1.451 | 0.537 |
